# Phenotype and Clinicoradiological Differences in Multifocal and Focal Bronchiectasis

**DOI:** 10.3390/medicina60050795

**Published:** 2024-05-10

**Authors:** Jelena Jankovic, Aleksandar Jandric, Natasa Djurdjevic, Dragan Vukosavljevic, Zlatan Bojic, Andrej Zecevic, Mihailo Stjepanovic

**Affiliations:** 1 Clinic for Pulmonology, University Clinical Center of Serbia, 11000 Belgrade, Serbia; jjelena1984@gmail.com (J.J.); jandricalexander@gmail.com (A.J.); natalidjurdjevic@yahoo.com (N.D.); gan_dra@live.com (D.V.); bojic.zlatan@gmail.com (Z.B.); zecevic92andrej@gmail.com (A.Z.); 2 Medical Faculty, University of Belgrade, 11000 Belgrade, Serbia

**Keywords:** bronchiectasis, phenotype, chest CT scan, hemoptysis, multifocal

## Abstract

*Introduction:* Bronchiectasis is a chronic progressive respiratory disease characterized by permanent dilatation of the bronchi. It is a complex condition with numerous different etiologies, co-morbidities, and a heterogeneous presentation. As we know, there is a lack of studies that describe the differences and compare the characteristics between focal and multifocal bronchiectasis. The aim of this study is to identify differences in clinical characteristics presentation, severity or distribution in focal and multifocal bronchiectasis, and prognostic implications. *Methods:* 126 patients with computed tomography (CT)-verified bronchiectasis were enrolled. Baseline characteristics that included age, sex, smoking history, and respiratory symptoms were recorded, with special attention paid to hemoptysis appearance, body mass index, and comorbidities. The type of bronchiectasis determined by CT scan and the modified Reiff scores indicating radiological severity were recorded. Patients were divided in two groups (I is focal and II is multifocal). *Results:* There were no statistically significant differences in age, smoking status, comorbidity, and BMI between the two groups. Multifocality was associated with a significantly higher proportion of females (*p* = 0.014), the rate of hemoptysis (*p* = 0.023), and the number of hospitalizations, but not of exacerbations and prevalence of immunodeficiency (*p* = 0.049). Significantly, a high number of subjects with multifocality had bronchiectasis of moderate severity, and post-infective and asthma-associated phenotypes were the dominant in this group. Unexpectedly, the cystic and varicose radiological phenotype (which need more time to develop) were more dominant in the focal group. The cylindrical phenotype was equally observed in the multifocal and focal groups. *Conclusions:* Our study suggests that multifocality is not related to age, number of exacerbations, or radiological phenotype, but it seems to be associated with the clinical post-infective phenotype, immunodeficiency, frequent hospitalizations, and severity. Thus, the presence of multifocal bronchiectasis may act as a biomarker of severity and poor outcomes in these patients.

## 1. Introduction

Bronchiectasis is a chronic progressive respiratory disease characterized by permanent damage and dilatation of the bronchi [1]. Bronchiectasis can be considered a syndrome and a common endpoint of various diseases, but it can also occur as a single radiological appearance that may or may not be associated with various diseases [2]. It is a complex condition with numerous different etiologies (infective, deficiency of alpha 1 antitrypsin, immunodeficiency, cystic fibrosis, and tuberculosis), co-morbidities (asthma and chronic obstructive pulmonary disease), and a heterogeneous presentation associated with respiratory symptoms and recurrent respiratory infections because of reduced mucus clearance, which favors mucus impaction [3]. The most common symptoms are cough, sputum production, hemoptysis, and fatigue. This clinical presentation is a consequence of the production of inflammatory mediators that lead to the destruction of elastin, affecting the muscular and cartilaginous part of the bronchus [4]. When local defense mechanisms are impaired, inflammation progresses, resulting in fibrosis and dilation of the bronchial wall and hyperinflation. This leads to a progressive decrease in pulmonary function parameters, which results in irreversible bronchodilation [5].

Previous epidemiological studies have largely considered bronchiectasis a rare disease, but the prevalence of bronchiectasis is increasing worldwide. It is now the third most common of all respiratory diseases [6]. A trend of increasing prevalence of bronchiectasis has been observed, with a rate of up to 566 per 100,000 persons [6,7]. Due to improved understanding and awareness, bronchiectasis with different clinical presentations is increasingly being reported. For a long time, bronchiectasis was an incidental radiological finding on chest computed tomography (CT) scans. Chest CT scans are now the gold standard in establishing the diagnosis of bronchiectasis, along with its staging, localization (in one or more lobes), and complications [8,9]. Radiological criteria for existing bronchiectasis include a broncho–arterial ratio of more than one, lack of bronchial tapering, or indirect signs such as peri-bronchial thickening, pulmonary nodules, mucus plugging, mosaicism, or air-trapping [1,9]. Thoracic scan imaging can establish the distribution pattern of bronchiectasis, supporting the severity assessment and long-term follow-up monitoring of its structural abnormalities and progression. 

The first classification of bronchiectasis is cystic fibrosis (CF)-related bronchiectasis or non-CF-related bronchiectasis. The Reid classification includes three main types of radiological phenotype of bronchiectasis: cylindrical, varicose, and cystic [10]. This classification describes cylindrical bronchiectasis as the mildest form of bronchiectasis with uniform caliber where there is a loss of tapering of the bronchus and dilatation of the bronchial lumen. As bronchiectasis progresses, it develops into varicose form bronchiectasis with irregularities in the bronchial wall and a distorted and significant shortening of the number of identifiable airway branches along with further dilatation. The most severe grade is cystic bronchiectasis with progressive saccular dilatation of the bronchial lumen terminating in a large cystic structure, often with increased air–fluid levels [10,11]. 

The distribution of bronchiectasis may also reflect the specific etiology. CT scans allow differentiation between focal (involving only one lobe) and diffuse or multifocal (involving two or more lobes) bronchiectasis [12]. Diffuse bronchiectasis develops most often in patients with genetic disorders, immunodeficiency, chronic diseases, or anatomical defects that affect the airways. Focal bronchiectasis develops as a result of untreated pneumonia or obstructions (due to foreign bodies or tumors) where the large airway is obstructed resulting in an inability to clear secretions and leading to a cycle of infection, inflammation, and airway wall damage. Multifocal bronchiectasis occurs when an underlying disorder triggers inflammation in the small- and medium-sized airways [13]. As for tumors, bronchiectasis multifocality surgical treatment is difficult. According to the European Respiratory Society (ERS) guidelines for the management of adult bronchiectasis, surgical treatment should not be considered until the maximum effect from all therapeutic options has been achieved [14]. For this reason, it is important to be aware of the characteristics and potential for multifocal bronchiectasis. The disease profile, prognosis, and outcome of treatment of bronchiectasis are different for each patient.

There is a lack of studies describing the differences and comparing the characteristics of focal and multifocal bronchiectasis. The aim of this study was to elucidate the differences in the clinical characteristics and presentation, severity, and radiological phenotype of focal and multifocal bronchiectasis.

## 2. Materials and Methods

### 2.1. Study Group and Data Collection

In this retrospective study, 126 patients with bronchiectasis who were treated at the Clinic for Pulmonology, University Clinical Center of Serbia, between 2020 and 2023 were included. This study was approved by the Local Committee in Belgrade and all the subjects gave signed informed consent, based on the principles of the International Declaration of Helsinki. 

The baseline characteristics of all participants, including age, sex, smoking history, respiratory symptoms (particularly hemoptysis appearance), weight, height, body mass index (BMI), and self-reported comorbidities (asthma, chronic obstructive pulmonary disease, diabetes mellitus, arterial hypertension, atrial fibrillation, rheumatic diseases, immunodeficiency, and others) were obtained from their electronic medical records. Exacerbations and hospitalizations were obtained by patients’ self-reports and validated through electronic medical records. Standard management in our center includes blood cell count; spirometry functional tests; detection of immunological factors; and sputum culture. The inclusion criteria were an age of over 18 years, a diagnosis of non-cystic fibrosis bronchiectasis via a computed tomography (CT) scan, and clinical symptoms consistent with bronchiectasis. 

The exclusion criteria were cystic fibrosis, active tuberculosis (TB), and active malignancy. 

### 2.2. Radiological Assessment

**Figure 1:**
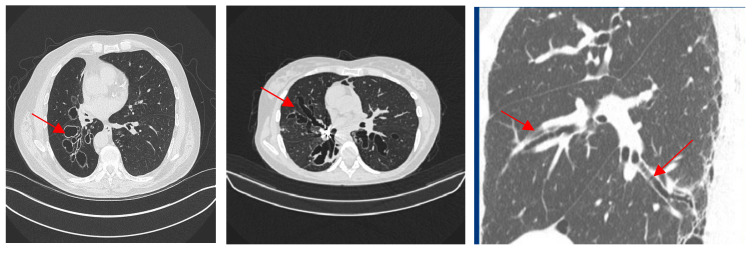
Three radiological types of bronchiectasis: (**a**) cystic, (**b**) varicose, and (**c**) cylindrical.

One chest radiologist evaluated the CT findings, the type of bronchiectasis, and the number of lobes affected by bronchiectasis. The CT findings were used to divide the participants into the focal (involving one lobe) and multifocal (involving two or more lobes) groups. The three radiological types of bronchiectasis are cylindrical, varicose, and cystic (Figure 1) [10]. The modified Reiff scores indicating the radiological severity of bronchiectasis were calculated [15]. This score takes into account the number of lobes involved (considering the lingual as a separate lobe) and the dilatation degree (cylindrical = 1, varicose = 2, and cystic = 3), to give a score between 1 and 18. The CT scoring system was as follows: mild bronchiectasis, 0–6; moderate, 7–12; and severe bronchiectasis, 13–18 [16]. The CT findings were evaluated by radiologist with more than 15 years of experience in the field, who worked at the Center for Radiology and Magnetic Resonance, University Clinical Center of Serbia.

### 2.3. Statistical Analysis

The analyses were performed in SPSS Statistics for Windows, version 21.0 (Chicago, USA). A *p* value < 0.05 was significant. Mean and standard deviation values were used for continuous variables and were compared using the *t*-test for independent samples. The Chi-squared test was used to compare categorical variables, and the data were presented as numbers with a percentage. A one-way ANOVA was used to compare the mild, moderate, and severe sub-scores of the modified Reiff score. The results are presented as graphics and tables.

## 3. Results

This study was a retrospective study of 126 patients with confirmed bronchiectasis, treated at the Clinic for Pulmonology, University Clinical Center of Serbia, for the period from 2019 to 2023. The mean age of the patients was 62.1 years. Nearly two-thirds of the patients were females, and the same percentage is found in the multifocal group. Nearly the same percentage of patients were non-smokers and former or current smokers. More than 80% of the patients in both groups had one or more comorbidities, the most frequent were obstructive diseases (asthma and COPD), with no statistically significant differences between groups. Other comorbidities, found in less than 10% of all patients, were diabetes mellitus, atrial fibrillation, nasal polyposis, and gastroesophageal reflux disease. Chronic rhinosinusitis was the most frequent comorbidity in the multifocal group (*p* = 0.006). Immunodeficiency was statistically more frequent in focal group (*p* = 0.049). In the focal group, patients were overweight and had higher values of BMI than in those of the multifocal group (26.3 vs. 23.2). But there were no statistically significant differences. The demographic characteristics are presented in Table 1.

**Figure 2:**
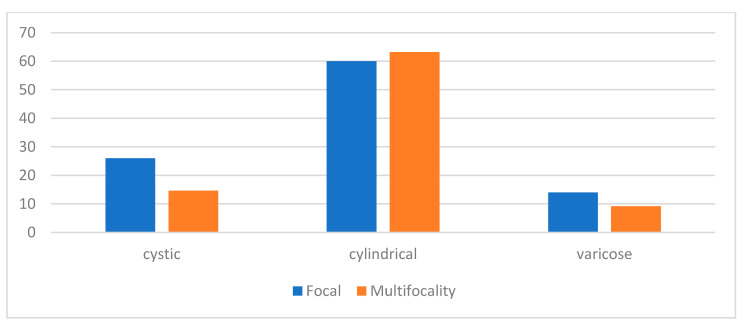
Prevalence of multifocality (orange) and focal (blue) in regard to specific radiological phenotypes of bronchiectasis.

Bronchiectasis was confirmed by chest CT scan. In 78 (61.9%) of the patients the cylindrical type was diagnosed. Varicose was diagnosed in 14 (11.1%), and cystic in 24 (19%). More than one type of bronchiectasis was observed in 48 subjects (38.1%). But there was difference in predominance in types between two groups: cystic (26 vs. 19.7%) and varicose (14 vs. 13.4%) in focal group, opposite then cylindrical (60 vs. 66.7%) in multifocal group. The prevalence of multifocality and focality with regards to specific radiological phenotypes of bronchiectasis is presented in Figure 2. 

Multifocality was associated with a significantly higher proportion of the female sex (*p* = 0.014), rate of hemoptysis (*p* = 0.023), the number of hospitalizations, and the prevalence of immunodeficiency (*p* = 0.049). 

All patients had typical respiratory symptoms such as fatigue, cough, and expectoration, but there was a difference in the frequency of hemoptysis with predominance in the multifocal group (*p* = 0.023). There were no statistically significant differences in the use of long-term oxygen treatment (LTOT) and the number of exacerbations in one year between the two groups. Patients with multifocal disease had a higher number of hospitalizations in one year (average of three or more). The clinical characteristics of the two groups are presented in Table 2.

According to the modified the Reiff score, 103 patients had mild bronchiectasis (81.8%), 10 patients had moderate bronchiectasis (7.9%), and 13 patients had severe bronchiectasis (10.3%). Only eight (6.3%) patients had multifocality on one side (left or right); the majority had it on both sides. Significantly, a higher number of cases with moderate severity was observed in the subjects with multifocality. Post-infective and asthma-associated phenotypes were dominant in the multifocal group of patients. 

Unexpectedly, the cystic radiological phenotype was more dominant in the focal group, and the cylindrical and varicose phenotypes were close to equal in both groups. The rate of cystic type (26% vs. 19.7%, *p* = 0.007) was significantly higher in patients with a focal pattern compared to patients with multifocality. The radiological presentation and phenotype differences between the two groups are presented in Table 3. 

## 4. Discussion

Multifocality is defined as the presence of multiple distinct disease foci and may have an impact on the management of the primary lesion and influence treatment decisions [17]. Multifocality mostly refers to tumors (breast and thyroid gland), but other lesions and changes, if not localized, can be classified using this term. Multifocal or diffuse bronchiectasis affect many areas of the lungs, with various different pathophysiological presentations [13]. 

We confirmed multifocality if bronchiectasis occurred in two or more lobes on the same or opposite side. Only 8 of 126 patients had multifocality on one side (left or right lung), the majority had bronchiectasis on both sides, predominantly in the lower lobes. This means that the underlying disease did not develop unilaterally; the aspiration of contents was usually on one side and tended to be more dominant on the right side due to the anatomical position of the right main bronchus [18]. The clinical post-infectious phenotype is a consequence of repeated infections and pneumonia and is also associated with asthma, which is relevant to the both-side theory. The association with asthma—considering the pathophysiology and mechanism of asthma—and the changes in the airways—which cannot occur in only one lung—explain the multifocal finding of bronchiectasis in this phenotype [19,20]. Bronchiectasis is the most frequent asthma-associated comorbidity according to the literature and GINA guidelines [20]. These two diseases have same etio-pathogenesis and common causes (such as gastroesophageal reflux disease, chronic rhinosinusitis, and allergic patterns) [19,21]. The concomitant presence of asthma and bronchiectasis (as a comorbidity or overlapping) is characterized by more severe respiratory symptoms, frequent exacerbations, functional decline, and worse outcomes [19,20,21]. In our study, rinosinusitis was significantly more frequent in multifocal bronchiectasis, which was also the most frequent clinical phenotype associated with asthma. Eosinophils are dominant inflammatory cells in asthma and rinosinusitis, causing inflammation and worsening of disease or remodeling. Failure to recognize asthma-associated bronchiectasis is often responsible for the worsening of symptoms and frequent exacerbations, along with greater disease severity. Bronchiectasis must be recognized and the most appropriate therapeutic approach selected in order to prevent poor outcomes [21]. In our study group, the patients with the asthma clinical phenotype had rhinosinusitis more frequently; the best treatment for this group is corticosteroids. The post-infective phenotype, depending on the frequency and localization of the infection itself, depends on whether a focal or diffuse form of bronchiectasis develops and was present in both groups [9]. The pathophysiology of focal bronchiectasis is usually the result of a local event, such as pneumonia [22]. This explains the most frequent post-infective phenotype in focal presentations. 

There were no differences in the frequency of the cylindrical radiological phenotype in the two groups. This is in line with the literature, which indicates that this is a dominant phenotype. The data also supported its presence in obstructive diseases and post-infectious phenotypes and its predominance in the lower lobes in these disorders [22,23]. Based on the radiological type and involvement of lobes and according to the modified Reiff score in our study, a significantly higher number of patients with moderate severity had multifocality. This may have been the reason for the statistically higher frequency of hospitalizations in this group of patients. We can conclude that the post-infectious phenotype, in moderate and severe form, frequent bilateral representation of the lower lobes, and LTOT use are related to the number of hospitalizations in patients with multifocal bronchiectasis.

Chronic respiratory failure is serious complication of bronchiectasis. According to previous studies, up to 40% of patients with bronchiectasis require LTOT at home because of asthma or chronic obstructive pulmonary disease (COPD). Bronchiectasis is present in 29–50% of COPD patients [24]. COPD was the main comorbidity in our study population. COPD and bronchiectasis can be related in three ways. COPD can lead to the development of bronchiectasis through airway inflammation and repeated bacterial infections [25]. Bronchiectasis in a non-smoker leads to a fixed airway obstruction and can progress to COPD [25,26]. Finally, COPD and bronchiectasis can be two independent and coexisting diseases [26]. Respiratory insufficiency was present in 13 of our patients with LTOT, and nearly 80% of them had history of COPD or pulmonary fibrosis. There was no difference between the groups in the use of LTOT because most of them were patients who had already been prescribed oxygen therapy at home. In both groups, there were patients with advanced COPD or fibrosis without a difference in the prevalence of these two diseases in both groups. 

The main respiratory symptoms in patients with bronchiectasis are cough, sputum expectoration, and breathlessness [27]. All of our patients had typical respiratory symptoms, although we focused on the frequency of hemoptysis in our study group. Hemoptysis was dominant in the multifocal group. This was typically not life-threatening hemoptysis; there was a need for embolization in two cases. Bronchiectasis is a frequent cause of hemoptysis, which can range from mild to massive hemoptysis [28]. In previous studies, hemoptysis occurred in 22–25% of bronchiectasis patients [29]. In a similar study performed in Germany, hemoptysis was a diagnosed in 15% of hospitalized patients with bronchiectasis [30]. Our result of 18.3% is in accordance with the literature. Male sex, smoking history (former or current), the lower right lobe, ≥2 involved lobes, cylindrical bronchiectasis, and COPD comorbidity were more likely to be associated with hemoptysis in our study population. Our study demonstrated that bronchiectasis patients with hemoptysis had less severe bronchiectasis (the Reiff score for all but two patients was six or lower). This is in accordance with a study by Seo and colleagues who also showed that bronchiectasis with hemoptysis had a lower bronchiectasis severity and short-term mortality [31]. The explanation for those results and ours—with lower severity scores—may be that the hemoptysis phenotype is not associated with severe bronchiectasis but with specific etiology and radiological findings. In our case, this is so with the cylindrical type, but in Seo’s study, this is so with the cystic type. If we are talking about etiology, in the abovementioned study, hemoptysis was associated with the infective phenotype (tuberculosis and mycetoma); also, in our both groups, the main clinical phenotype is post-infective. 

Bronchiectasis can occur at any age, but the highest prevalence is in older women [32]. Only a few studies have described sex differences in patients with bronchiectasis. Women have a different lung physiology: their lungs are smaller, and they have smaller conducting airways, with pseudostratified ciliated epithelial tissue with mucus-secreting properties. Continuous chronic inflammation can potentially contribute to greater lung tissue damage and worse disease severity [32,33]. Female sex was dominant in our bronchiectasis study population and was also more frequent in the focal group than the multifocal group.

This study had several limitations. First, as with any retrospective study, our data were dependent on the availability of medical history and records. This was a single-center study, so geographical differences could not be assessed. A large, multicenter study is needed. The amount of expectorated blood was determined from the medical records, so there may have been some limitations in assessing hemoptysis severity. Finally, not all patients had their BMI measured.

## 5. Conclusions

The results of our research indicated that, compared with a focal presentation, multifocality should be regarded as a significant prognostic factor for clinically severe disease, especially in patients with immunodeficiency, frequent hospitalizations, and hemoptysis, which can sometimes be life-threatening and is associated with a decreased quality of life. Our data also suggested that the presence of multifocality is associated with clinical post-infective and asthma related phenotypes. Identification of distinct phenotypes and the spread of the disease will lead to greater insight into the characteristics, treatment, and outcomes of bronchiectasis, as bronchiectasis is often underreported and underdiagnosed. Appropriate management to reduce the disease burden associated with poor quality of life is needed in patients with bronchiectasis.

## Figures and Tables

**Table 1:** Demographic characteristics between the two groups.

	Focal	Multifocal	*p*
N (%)	50 (39.7)	76 (60.3)	
Age years	62.8 ± 7.1	61.4 ± 6.4	0.762
Gender N (%) m/f	17 (34)/41 (66)	36 (47.4)/40 (52.6)	0.014
Smoking N (%) no/yes	23 (46)/27 (54)	41 (53.9)/35 (46.1)	0.065
BMI	26.3	23.3	0.062
Immunodeficiency N (%)	8 (16)	10 (13.1)	0.049
Comorbidity N (%)	46 (92)	63 (82.9)	0.121
Chronic rhinosinusitis	12 (24)	17 (46)	0.006
COPD	17 (34)	29 (38)	0.119
Asthma	15 (30)	22 (28.9)	0.237

**Table 2:** Clinical characteristics between two groups.

	Focal	Multifocal	*p*
N (%)	50 (39.7)	76 (60.3)	
Hemoptysis	8 (16)	15 (19.7)	0.023
≥2 hospitalizations in 1 year N (%)	3 (12)	17 (22.4)	0.011
LTOT N (%)	5 (10)	8 (10.5)	0.665
≥2 exacerbations in 1 year N (%)	11 (22)	18 (23.7)	0.073

**Table 3:** Radiological presentation and phenotypes differences between the two groups.

	Focal	Multifocal	*p*
Type N(%)			
Cystic	13 (26)	15 (19.7)	0.007
Cylindrical	30 (60)	50 (66.7)	0.126
Varicose	7 (14)	11 (13.4)	0.331
Localization N (%)			
Left	25 (50)	2 (2.6)	0.974
Right	25 (50)	6 (7.9)	0.893
Bilateral	N\A	68 (89.5)	
Severity N(%)			
Mild	49 (98)	54 (71.1)	0.017
Moderate	1 (2)	9 (11.8)	0.009
Severe	0	13 (17.1)	0.003
Clinical phenotype	Post-infective	Post-infective Asthma	

## Data Availability

The data that support the findings of this study are available from the corresponding author (M.S.) upon reasonable request.
